# Development of an ELISA to detect early local relapse of colorectal cancer.

**DOI:** 10.1038/bjc.1989.308

**Published:** 1989-10

**Authors:** L. G. Durrant, H. Jones, N. C. Armitage, R. W. Baldwin

**Affiliations:** Cancer Research Campaign Laboratory, University of Nottingham, UK.

## Abstract

The Y haptenic blood group determinant is expressed on the cells of malignant gastrointestinal tumours and on the normal gastrointestinal epithelium of individuals who can secrete blood group antigens. an enzyme-linked immunosorbent assay incorporating the high affinity biotin-avidin interaction was developed to measure the serum levels of antigens bearing the Y hapten in patients with colorectal cancer. The specificity of the assays was between 88 and 93% and the sensitivity in detecting extensive disease was between 24 and 34%. However, up to 67% of patients with local or abdominal recurrent disease secreted antigens expressing the Y hapten, whereas only 30% of patients with overt hepatic metastases secreted a similar antigen. Recognition of antigens bearing the Y hapten may therefore be useful in detecting early local relapse of colorectal cancer when a second operation to excise the recurrence may be possible.


Br. J. Cancer (1989), 60, 533–537 © The Macmillan Press Ltd., 1989

### Development of an ELISA to detect early local relapse of colorectal cancer

L.G. Durrant[1], H. Jones[1], N.C. Armitage[2] & R.W. Baldwin[1]

[1]*Cancer Research Campaign Laboratories, University of Nottingham, Nottingham NG7 2RD; and* [2]*Department of Surgery, University Hospital, Queens Medical Centre, Nottingham NG7 2UH, UK.*

**Summary** The Y haptenic blood group determinant is expressed on the cells of malignant gastrointestinal tumours and on the normal gastrointestinal epithelium of individuals who can secrete blood group antigens. An enzyme-linked immunosorbent assay incorporating the high affinity biotin–avidin interaction was developed to measure the serum levels of antigens bearing the Y hapten in patients with colorectal cancer. The specificity of the assays was between 88 and 93% and the sensitivity in detecting extensive disease was between 24 and 34%. However, up to 67% of patients with local or abdominal recurrent disease secreted antigens expressing the Y hapten, whereas only 30% of patients with overt hepatic metastases secreted a similar antigen. Recognition of antigens bearing the Y hapten may therefore be useful in detecting early local relapse of colorectal cancer when a second operation to excise the recurrence may be possible.

There is considerable interest in non-invasive techniques for the identification of patients with recurrent disease or liver metastases following resection of primary colorectal tumours. At present carcinoembyronic antigen (CEA) is used in postoperative monitoring, in an attempt to detect early relapse. However, Finlay and McArdle (1983) showed recurrence to be identified by computerised tomography 7.5 months before any increase in CEA was seen. There is an urgent requirement for detection of a new tumour associated antigen which is secreted by a large percentage of early recurrent tumours, or for identification of an antigen which would complement CEA monitoring of recurrent disease.

The difucosylated type 2 carbohydrate chain associated with the H blood group antigen is referred to as the Y hapten. The monoclonal antibody C14, raised against extranuclear membranes of a human colonic adenoma has been shown to define the Y hapten (Brown *et al.*, 1983). Using C14, Brown *et al.* (1984) showed that 96% of colorectal adenocarcinomas and 100% of adenomas expressed the Y hapten. The expression was greater than that shown by any normal colonic mucosa (Durrant *et al.*, 1986). Although Y hapten can be detected in saliva from individuals who are blood group secretors, it is not known whether antigens bearing the Y hapten are shed into the circulation. We have developed a rapid accurate enzyme-linked immunosorbent assay to screen for antigen bearing Y hapten in sera.

### Materials and methods

### *Monoclonal antibody*

C14 is an IgM monoclonal antibody which was purified from mouse ascitic fluid on lentil lectin sepharose as previously described (Takacs & Staehelin, 1981) and stored at a concentration of 3.07 mg ml$^{-1}$ at -70°C. NCRC-21 is an IgM monoclonal antibody which was raised against a colorectal tumour glycolipid extract. It was subsequently found to react with an epitope on a high molecular weight mucin which also expressed the Y hapten (M.R. Price, unpublished results). It was purified from mouse ascitic fluid on lentil lectin sepharose as described above.

### *Immunoflourescence*

The activity of the biotinylated C14 was tested by indirect immunofluorescence on the colorectal cell line C170. Briefly, $2 \times 10^6$ cells were incubated at 4°C for 30 min with antibody (10 μg) before washing and incubating with either FITC-rabbit anti-mouse immunoglobulins (Dakopatts, Denmark) or FITC streptavidin (Serotech, Bicester, UK). Both incubations were for 30 min at 4°C. The cells were washed before analysis on a FACS IV using similar conditions to those previously described (Durrant *et al.*, 1986).

### *Biotinylation of C14*

Incubation of C14 monoclonal antibody with varying concentrations (30–120 μg ml$^{-1}$) of *N*-hydroxysuccinamide (NHS)-biotin (Guesden *et al.*, 1979) for 1 h at 4°C in the dark resulted in complete loss of antigen–antibody binding (Table I).

Conjugation of certain macromolecules to NHS-biotin may cause steric hindrance of the biotin–avidin interaction. The use of a spacer arm limits this problem. C14 was therefore labelled using a long chain derivative of NHS-biotin with a 13 atom spacer, arm, sulphosuccinimidyl-6-(biotin amindo)hexanote (NHS-LC-biotin; Pierce, Illinois, USA).

Freshly prepared NHS-LC biotin was dissolved in dimethylformamide (2 mg ml$^{-1}$) and added (60–120 μg per 1 mg of antibody) to C14 monoclonal antibody in sodium bicarbonate buffer at pH 9.0. The reaction was stirred gently in the dark for 1 h. Biotinylated antibody was separated from free NHS-LC-biotin on a PD-10 column, when NHS-LC-biotin was used at concentration of 60 μg ml$^{-1}$ it resulted in retention of antibody binding (Table I) and good levels of biotinylation (Table I). The lower mean fluorescence obtained using streptavidin-FITC compared to rabbit anti-mouse-FITC were due to differing binding ratios. Each biotin molecule can bind only one streptavidin FITC whereas each mouse antibody can bind two to three rabbit anti-mouse antibodies. There are two to three molecules of fluorescein

**Table I** Indirect immunofluorescence of biotinylated C14 on C170 colorectal cancer cells

| *Primary antibody*[a] | *Mean fluorescence* | |
|---|---|---|
| C14 | 880[b] | 7[c] |
| nMse IgM | 11 | 6 |
| NHS-biotinylated | | |
| C14b (30)[d] | 14 | 12 |
| C14b (60) | 10 | 14 |
| C14b (120) | 20 | 8 |
| NHS-LC-biotinylated | | |
| C14b (60) | 403 | 192 |
| C14b (120) | 87 | 12 |

[a]Primary antibody (10 μg per $2 \times 10^6$ cells) as specified. [b]Secondary antisera was FITC-conjugated Rabbit anti-mouse (1.40). [c]Secondary reagent streptavidin-FITC (1:400). [d]Figures in parenthesis refer to concentrations of NHS-biotin or NHS-LC biotin which were used in the original biotinylations.

Correspondence: L.G. Durrant.
Received 20 October 1988; and in revised form 19 April 1989.

isothiocyanate attached to each streptavidin or antibody molecule. As the mean fluorescence obtained with C14 biotin recognised by FITC conjugate rabbit anti-mouse sera was twice the value obtained with streptavidin FITC it suggests that only one biotin molecule per C14 antibody molecule was available to recognise streptavidin-FITC. Increasing the dose of NHS-LC-biotin to attach more molecules of biotin resulted in a large loss of binding activity (Table I) as did increasing the incubation time (data not shown).

### *ELISA to detect C14 defined antigen*

C14 (500 ng per well) or NCRC-21 (500 ng per well) was added to flexible microtest plates (Falcon, Becton Dickinson, Oxnoid, CA, USA) and left at 4°C overnight. The plates were washed three times with phosphate buffered saline containing 0.05% Tween 20 (PBS-Tween) and blocked with BSA (10%) for 1 h at room temperature. The plate was washed three times, then serial dilutions of saliva or sera in a buffer at pH 3.0 (5 g BSA, 50 nmol sodium citrate, 1 mmol EDTA in 1 litre of $H_2O$) were added. After 1 h at room temperature the plates were washed five times in PBS-Tween and biotinylated C14 (500 ng per well) was added. Following incubation at room temperature for 1 h and washing five times, avidin alkaline phosphatase (Sigma, UK) diluted 1:400 was added for a further 1 h. Finally, after extensive washing the plates were developed with *P*-nitrophenol phosphate (Sigma, UK) and read at 405 nm.

### *Radioimmunoassays for the detection of carcinoembryonic antigen and CA19-9 antigen*

Carcinoembryonic antigen (CEA) was estimated using a commercially available radioimmunoassay kit (Serona Labs) based on a polyclonal antibody to CEA. Ten $\mu g\,l^{-1}$ was defined as positive. CA19-9 was measured using a commercially available solid phase radioimmunoassay (CIS UK Ltd, London). Thirty-three units $ml^{-1}$ was defined as positive.

### *Sera samples*

The sera of 55 patients with extensive colorectal carcinomas were tested for the presence of an antigen bearing the Y hapten. Of these patients, 36 had liver metastases, one had lung and brain metastases and 15 had pelvic or abdominal recurrent disease including five patients who had both liver metastases and recurrence and three had second primaries. Forty-three samples from normal subjects with no bowel symptoms were also screened.

The absorbence at 405 nm ($A_{405}$) of the positive results was converted into units $ml^{-1}$ as follows using the saliva from one secretor as a standard.

$$A_{405}\text{ standard saliva} - A_{405}\text{ buffer alone} = 10\text{ units (under constant conditions)}$$

$$\frac{A_{405}\text{ sample} - A_{405}\text{ buffer}}{A_{405}\text{ standard saliva} - A_{405}\text{ buffer alone}} \times 10\text{ units in }50\,\mu l$$

Units in 50 $\mu l \times 20$ = units of Y haptenic blood group determinant $ml^{-1}$. Buffer alone plus two standard deviations never exceeded 20 units $ml^{-1}$ for detection by C14 biotin and 50 units $ml^{-1}$ for detection by NCRC-21 biotin. These levels were therefore the values for positive results.

### Results

### *Development of the ELISA for detection of C14*

The ELISA assays depend upon either recognition of the Y hapten on an antigen by the solid phase capturing C14 antibody or recognition of the NCRC-21 defined epitope on an antigen and then recognition of the Y hapten on these antigens by the biotinylated C14. Initial assays performed on saliva from normal individuals including blood group secretors confirmed that antigens bearing two Y haptens or an NCRC-21 epitope and a Y hapten existed (Figure 1). However, when the serum from normal individuals was screened (Figure 1), sera from 3/43 (7%) normal donors gave a positive result with the C14 capturing antibody (Figure 1). Only one of these positive sera was from an individual with antigen in his saliva.

**Figure 1** Expression of an antigen expressing the Y haptenic blood group in sera and saliva of normal individuals as recognised by the antibodies C14(*a*) and NCRC-21(*b*). The line represents the mean units + 2 standard deviations of buffer alone and has been used for defining positive values.

Sera from 34% of the patients with colorectal cancer gave positive results when C14 was the capturing antibody (Figure 2). However, 67% of the patients with pelvic or abdominal recurrent disease secreted antigen bearing the Y hapten into their serum compared with 30% of patients with metastases were positive ($\chi^2 = 4.6; 0.05 > P > 0.02$). Five patients had both liver metastases and recurrent disease. Of these three showed positive results and two were negative. If these patients are excluded from the total patient analysis 70% of patients with only recurrent disease and 28% of patients with metastases alone are positive. Antigen captured by NCRC-21 monoclonal antibody was detected in 24% of patients sera. Thirty-three per cent of patients with recurrent disease expressed this antigen and 22% of patients with metastases (Figure 2).

There was no correlation between expression of the antigen(s) captured by C14 and those captured by NCRC-21 (Figure 3). Seven patients secreted an antigen expressing two Y haptenic determinants but no NCRC-21 defined antigen whereas one patient expressed the NCRC-21 defined antigen but did not express two accessible Y haptenic determinants. Furthermore, there was no correlation between the expression of CEA, CA19-9 and the two types of antigen defined in these assays (Figure 3).

CEA was still the most sensitive monitor of metastatic

**Figure 2** Expression of an antigen expressing the Y haptenic blood group in a sera of patients with colorectal cancer as recognised by the antibodies C14(*a*) and NCRC-21(*b*). P, all patients tested; M, patients with overt liver metastases; R, patients with recurrent disease. The line represents the mean units + 2 standard deviations of buffer alone and has been used for defining positive values.

**Figure 3** Correlations between the levels of antigen expressing the Y haptenic blood group in sera of patients with colorectal cancer and the levels of carcinoembryonic antigen (CEA) and levels of CA19-9 antigen.

disease as 73% of these patients had elevated levels of this antigen. The sensitivity of detection of recurrent disease is similar for CEA and C14 assays. However, detection of both recurrence and metastatic disease can be increased to 80% and 82%, if CEA assays are combined with C14 and NCRC-21 ELISAs. Similarly, the sensitivity of the CA19-9 assay in detecting recurrent disease can be doubled by also screening by C14 and NCRC-21 ELISAs (Table II).

**Table II** Sensitivities of the tests for CEA, CA19-9 and Y hapten (using C14 and NCRC-21 as the catching antibody)

| Test | *All sera* | | *Sera from recurrent patients* | | *Sera from patients with metastases* | |
|---|---|---|---|---|---|---|
| | *+ve*[a] | *S%*[b] | *+ve* | *S%* | +ve | S% |
| C14 | 19/55 | 34 | 10/15 | 67 | 6/22 | 27 |
| NCRC-21 | 13/55 | 24 | 5/15 | 33 | 4/22 | 18 |
| CEA | 23/36 | 64 | 10/15 | 67 | 16/22 | 73 |
| CA19-9 | 14/35 | 40 | 6/15 | 40 | 11/22 | 50 |
| C14 & NCRC-21 | 21/55 | 38 | 10/15 | 67 | 7/22 | 32 |
| CEA & CA19-9 | 26/35 | 74 | 11/15 | 73 | 18/22 | 82 |
| C14 & NCRC-21 & CEA | 28/36 | 78 | 12/15 | 80 | 18/22 | 82 |
| C14 & NCRC-21 CA19-9 | 23/36 | 64 | 12/15 | 80 | 13/22 | 59 |
| C14 & NCRC-21 & CEA & CA19-9 | 29/36 | 81 | 12/15 | 80 | 19/22 | 86 |

[a] + ve, percentage of samples positive. [b]S%, sensitivity.

### Discussion

The Y haptenic blood group determinant is known to be expressed by malignant gastrointestinal cells (Brown *et al.*, 1984; Lloyd *et al.*, 1983; Abe *et al.*, 1986). This project shows that it is also released into the circulation in up to 33% of patients with extensive colorectal cancer.

RIA have previously been used in the detection of circulating tumour-associated antigens. The ELISA developed in this study presents many advantages over the RIA. Since no radioisotopes are used the ELISA is less hazardous and can be perfomred in any laboratory without the need for expensive gamma-counters. Radiolabelled antibody remains stable for only 1–2 months, whereas biotinylated antibody can be stored for an indefinite period. A large batch of antibody can be biotinylated and used in numerous assays, thereby allowing results to be reproducible.

Brown *et al.* (1984) showed that the Y hapten was strongly expressed on the normal gastrointestinal epithelia of individuals of secretor status while being weak or absent in non-secretors. Of the normal individuals tested 11/13 were secretors but only one of these expressed Y hapten bearing antigens in the serum. It would appear that the antigen is not released generally into the circulation from normal gastrointestinal cells. All antigens detected by the assays in this study must carry a Y haptenic blood group determinant. There may, however, be several discrete antigens carrying this specific epitope. An antigen carrying a minimum of two Y haptenic blood group determinant and an epitope recognised by NCRC-21 would give a positive result using NCRC-21 as the catching antibody. A third possibility is that in addition to there being these two different types of antigen, there is a third type which carries both an NCRC-21 epitope and two or more Y haptenic blood group determinants. Seven patients secreted an antigen recognised by two C14 antibody molecules but failed to bind to NCRC-21 and one patient secreted an antigen which bound to NCRC-21 and C14 but not to two C14 antibody molecules. These results suggest that at least two discrete antigens are being detected by the ELISA assays.

Thirty-four per cent of colorectal cancer patients with extensive disease secreted an antigen recognised by two C14 molecules and 24% an antigen recognised by C14 and NCRC-21. This sensitivity is low compared to CEA (64%) but similar to that seen for secretion of CA19-9 antigen (40%). However, there was no obvious correlation between expression of any of the antigens and in fact co-screening for all four antigens improved the sensitivity of recurrent cancer detection to 80%.

C14 shows a sensitivity of 67% for patients with recurrence and only a 30% sensitivity for patients with metastases, whereas CEA showed a similar sensitivity for both groups of patients and CA19-9 showed an elevated sensitivity in patients with overt hepatic metastases. This suggests that C14 could prove to be of clinical value in detecting recurrence that is potentially curable by second operations. This is particularly important in view of the fact that serum CEA levels are related to tumour bulk and therefore raised levels often occur when recurrence is beyond cure and liver metastases are present (Finlay & McArdle, 1983). Despite a number of encouraging reports showing CEA to have a high sensitivity for recurrence (reviewed by Northover, 1986), its late stage expression (Finlay & McArdle, 1983) and its high false negative rate of approximately 20% (Herrera *et al.*, 1976; Wood *et al.*, 1980) suggest its clinical value in detecting early recurrences is limited.

A further study including serum samples from a large age-matched normal panel, patients with inflammatory diseases, ademomatous polyps, primary cancer, recurrent disease and liver metastases is currently in progress. A prospective study to determine if this assay can detect recurrence earlier than other methods and hence improve treatment is also ongoing. However, these results are encouraging in view of the fact that no other tumour antigen has been reported to be expressed more by recurrent disease than by liver metastases. Antigens expressing the Y hapten may be useful in identifying patients with early potentially curable recurrent disease.

This work was funded by a grant from the Cancer Research Campaign.

### References

ABE, K., HAKOMORI, S. & OHABIBA, S. (1986). Differential expression of difucosyl Type 2 chain (le$^Y$) defined by monoclonal antibody AH6 in different locations of colonic epithelia, various histological types of colonic polyps and adenocarcinomas. *Cancer Res.*, **46**, 2639.

BROWN, A., ELLIS, I.O., EMBLETON, M.J., BALDWIN, R.W., TURNER, D.R. & HARDCASTLE, J.D. (1984). Immunohistochemical localization of Y hapten and structurally related H type-2 blood group antigen on large bowel tumours and normal adult tissues. *Int. J. Cancer*, **33**, 727.

BROWN, A., FEIZI, T., GOOI, H.C., EMBLETON, M.J., PICARD, J.K. & BALDWIN, R.W. (1983). A monoclonal antibody against human colonic adenoma recognizes difucosylated type-2 blood group chains. *Biosci. Rep.*, **3**, 163.

DURRANT, L.G., ROBINS, R.A., ARMITAGE, N.C., BROWN, A., BALDWIN, R.W. & HARDCASTLE, J.D. (1986). Association of antigen expression and DNA ploidy in human colorectal tumour. *Cancer Res.*, **46**, 3543.

FINLAY, I.G. & McARDLE, C.S. (1983). Role of carcinoembryonic antigen in detection of asymptomatic disseminated disease in colorectal carcinoma. *Br. Med. J.*, **288**, 1242.

GUESDEN, J.-L. TERNYNK, T. & AVRAMEAS, S. (1979). The use of avidin-biotin interaction in immunoenzymatic techniques. *J. Histochem. Cytochem.*, **27**, 1131.

HERRERA, M.A., CHU, T.M. & HARDCASTLE, J.D. (1976). Carcinoembryonic antigen (CEA) as a prognostic and monitoring test in clinically complete resection of colorectal carcinomas. *Ann. Surg.*, **183**, 5.

LLOYD, K.O., LARSON, G., STROMBERG, N., THURIN, J. & KARLSSON, K.A. (1983). Mouse monoclonal antibody F-3 recognises difucosyl type-2 blood group structure. *Immunogenetics*, **17**, 537.

NORTHOVER, J. (1986). Carcinoembryonic antigen and recurrent colorectal cancer. *Gut*, **27**, 117.

TAKACS, B. & STAEHELIN, T. (1981). Biochemical characterisation of cell surface antigens using monoclonal antibodies. In *Immunological Methods, vol. 2*, Lefkovits, I. & Pernis, B. (eds) p.27. Academic Press: New York.

WOOD, C.B., RATCLIFFE, J.G., BURT, R.W., MALCOLM, A.J.H. & BLUMGART, L.H. (1980). The clinical significance of the pattern of elevated serum carcinoembryonic antigen (CEA) levels in recurrent colorectal cancer. *Br. J. Surg.*, **67**, 46.


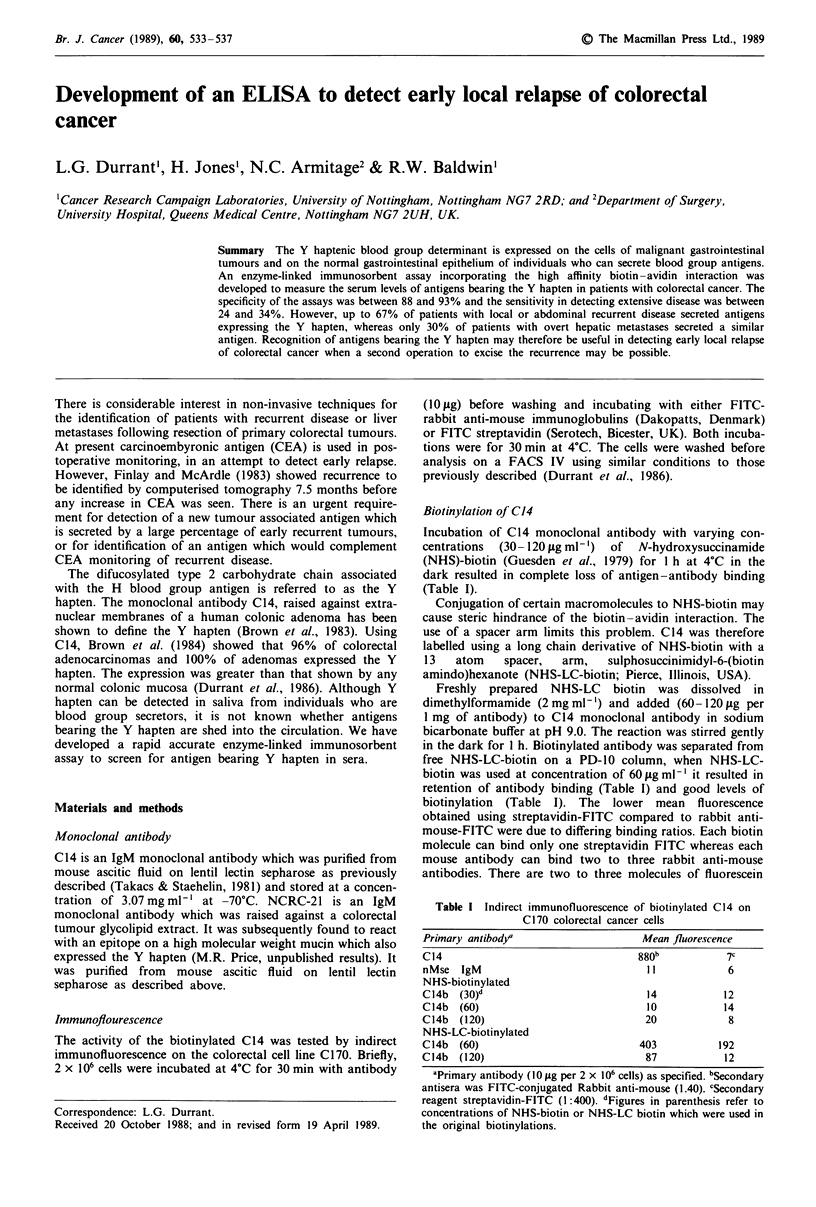

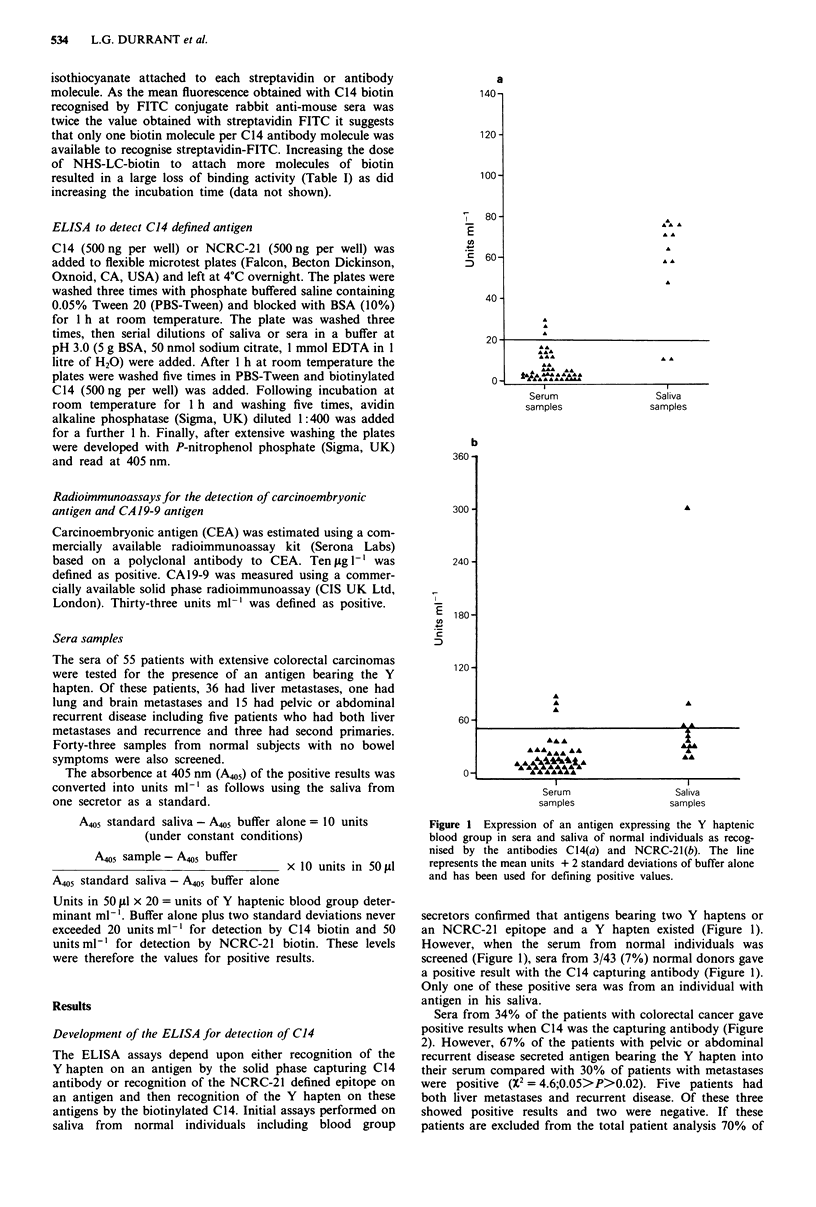

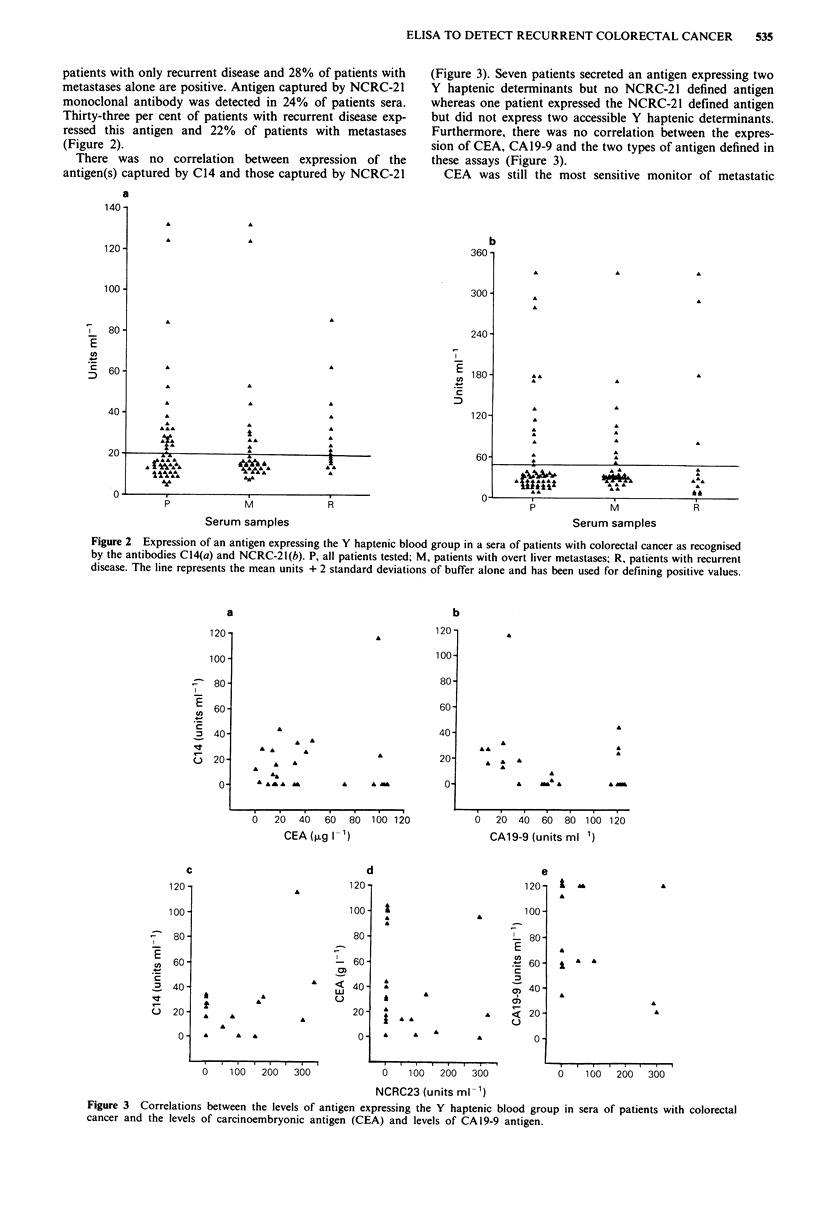

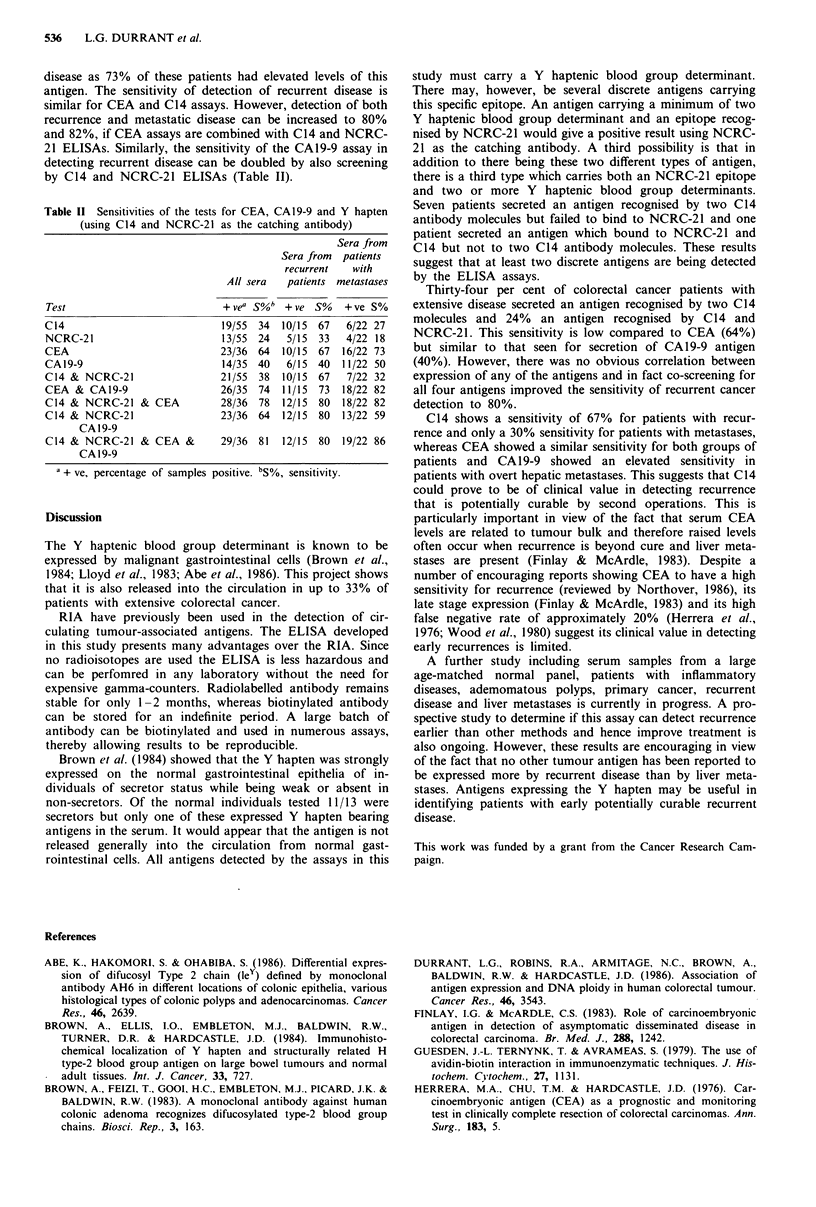

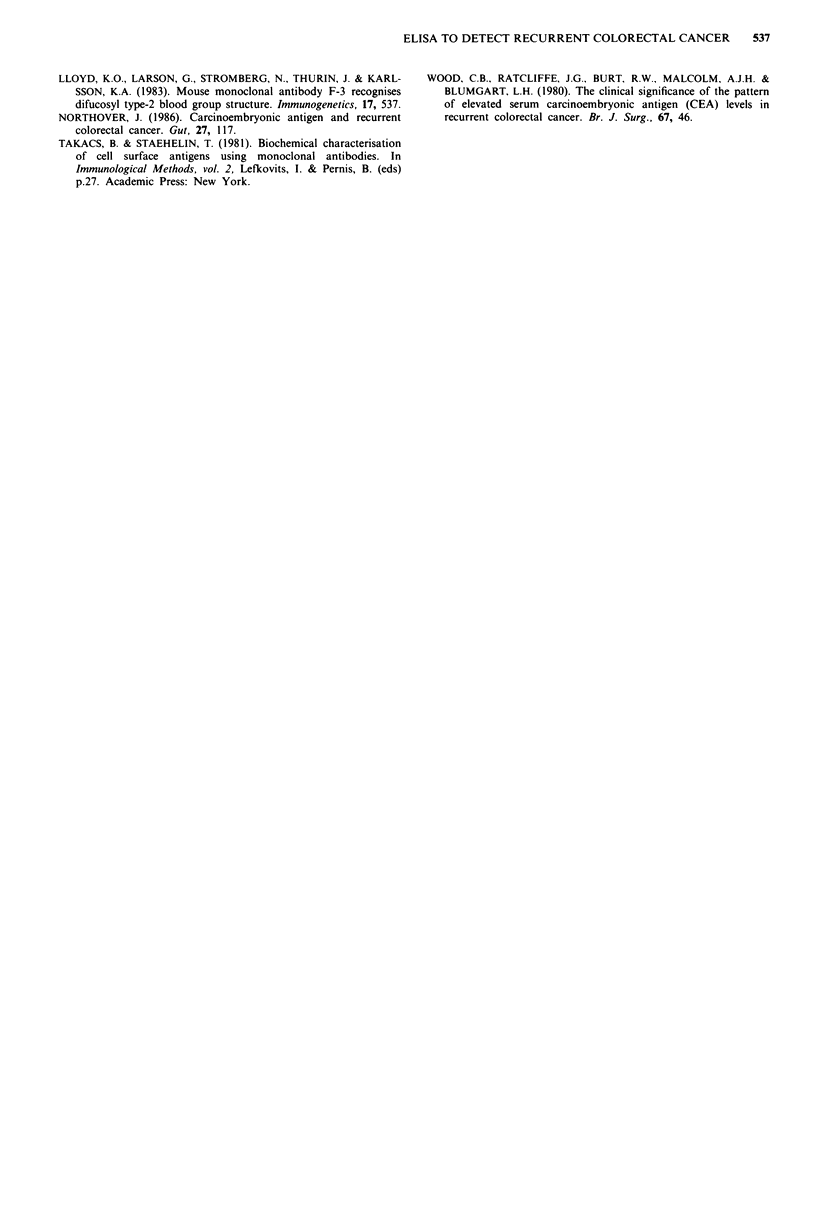


## References

[OCR_00741] Abe K., Hakomori S., Ohshiba S. (1986). Differential expression of difucosyl type 2 chain (LeY) defined by monoclonal antibody AH6 in different locations of colonic epithelia, various histological types of colonic polyps, and adenocarcinomas.. Cancer Res.

[OCR_00748] Brown A., Ellis I. O., Embleton M. J., Baldwin R. W., Turner D. R., Hardcastle J. D. (1984). Immunohistochemical localization of Y hapten and the structurally related H type-2 blood-group antigen on large-bowel tumours and normal adult tissues.. Int J Cancer.

[OCR_00755] Brown A., Feizi T., Gooi H. C., Embleton M. J., Picard J. K., Baldwin R. W. (1983). A monoclonal antibody against human colonic adenoma recognizes difucosylated Type-2-blood-group chains.. Biosci Rep.

[OCR_00761] Durrant L. G., Robins R. A., Armitage N. C., Brown A., Baldwin R. W., Hardcastle J. D. (1986). Association of antigen expression and DNA ploidy in human colorectal tumors.. Cancer Res.

[OCR_00767] Finlay I. G., McArdle C. S. (1983). Role of carcinoembryonic antigen in detection of asymptomatic disseminated disease in colorectal carcinoma.. Br Med J (Clin Res Ed).

[OCR_00772] Guesdon J. L., Ternynck T., Avrameas S. (1979). The use of avidin-biotin interaction in immunoenzymatic techniques.. J Histochem Cytochem.

[OCR_00777] Herrera M. A., Chu T. M., Holyoke E. D. (1976). Carcinoembryonic antigen (CEA) as a prognostic and monitoring test in clinically complete resection of colorectal carcinoma.. Ann Surg.

[OCR_00787] Lloyd K. O., Larson G., Strömberg N., Thurin J., Karlsson K. A. (1983). Mouse monoclonal antibody F-3 recognizes the difucosyl type-2 blood group structure.. Immunogenetics.

[OCR_00789] Northover J. (1986). Carcinoembryonic antigen and recurrent colorectal cancer.. Gut.

[OCR_00799] Wood C. B., Ratcliffe J. G., Burt R. W., Malcolm A. J., Blumgart L. H. (1980). The clinical significance of the pattern of elevated serum carcinoembryonic antigen (CEA) levels in recurrent colorectal cancer.. Br J Surg.

